# The Value of Preoperative Local Symptoms in Prognosis of Upper Tract Urothelial Carcinoma After Radical Nephroureterectomy: A Retrospective, Multicenter Cohort Study

**DOI:** 10.3389/fonc.2022.872849

**Published:** 2022-06-02

**Authors:** Hsin-Chih Yeh, Chao-Hsiang Chang, Jen-Kai Fang, I-Hsuan Alan Chen, Jen-Tai Lin, Jian-Hua Hong, Chao-Yuan Huang, Shian-Shiang Wang, Chuan-Shu Chen, Chi-Wen Lo, Chih-Chin Yu, Jen-Shu Tseng, Wun-Rong Lin, Yeong-Chin Jou, Ian-Seng Cheong, Yuan-Hong Jiang, Chung-You Tsai, Thomas Y. Hsueh, Yung-Tai Chen, Hsu-Che Huang, Yao-Chou Tsai, Wei-Yu Lin, Chia-Chang Wu, Po-Hung Lin, Te-Wei Lin, Wen-Jeng Wu

**Affiliations:** ^1^ Department of Urology, Kaohsiung Municipal Ta-Tung Hospital, Kaohsiung, Taiwan; ^2^ Department of Urology, School of Medicine, College of Medicine, Kaohsiung Medical University, Kaohsiung, Taiwan; ^3^ Graduate Institute of Medicine, College of Medicine, Kaohsiung Medical University, Kaohsiung, Taiwan; ^4^ Department of Urology, Kaohsiung Medical University Hospital, Kaohsiung, Taiwan; ^5^ Department of Urology, China Medical University, Taichung, Taiwan; ^6^ School of Medicine, China Medical University, Taichung, Taiwan; ^7^ Department of Urology, China Medical University Hospital, Taichung, Taiwan; ^8^ Division of Urology, Department of Surgery, Kaohsiung Veterans General Hospital, Kaohsiung, Taiwan; ^9^ Institute of Biomedical Engineering, National Taiwan University, Taipei, Taiwan; ^10^ Department of Urology, National Taiwan University Hospital, College of Medicine, National Taiwan University, Taipei, Taiwan; ^11^ Division of Urology, Department of Surgery, Taichung Veterans General Hospital, Taichung, Taiwan; ^12^ Institute of Medicine, Chung Shan Medical University, Taichung, Taiwan; ^13^ Department of Applied Chemistry, National Chi Nan University, Nantou, Taiwan; ^14^ Department of Senior Citizen Service Management, National Taichung University of Science and Technology, Taichung, Taiwan; ^15^ Division of Urology, Department of Surgery, Taipei Tzu Chi Hospital, The Buddhist Medical Foundation, New Taipei City, Taiwan; ^16^ School of Medicine, Buddhist Tzu Chi University, Hualien, Taiwan; ^17^ Department of Urology, MacKay Memorial Hospital, Taipei, Taiwan; ^18^ Mackay Medical College, New Taipei City, Taiwan; ^19^ Institute of Biomedical Informatics, National Yang Ming Chiao Tung University, Taipei, Taiwan; ^20^ Department of Urology, Ditmanson Medical Foundation Chiayi Christian Hospital, Chiayi, Taiwan; ^21^ Department of Health and Nutrition Biotechnology, Asian University, Taichung, Taiwan; ^22^ Department of Urology, Hualien Tzu Chi Hospital, Buddhist Tzu Chi Medical Foundation and Tzu Chi University, Hualien, Taiwan; ^23^ Division of Urology, Department of Surgery, Far Eastern Memorial Hospital, New Taipei City, Taiwan; ^24^ Department of Healthcare Information & Management, Ming Chuan University, Taoyuan, Taiwan; ^25^ Division of Urology, Department of Surgery, Taipei City Hospital renai branch, Taipei, Taiwan; ^26^ Department of Urology, School of Medicine, National Yang Ming Chiao Tung University, Taipei, Taiwan; ^27^ Department of Urology, Taiwan Adventist Hospital, Taipei, Taiwan; ^28^ Division of Urology, Department of Surgery, Cardinal Tien Hospital, New Taipei City, Taiwan; ^29^ Department of Life Science, College of Science, National Taiwan Normal University, Taipei, Taiwan; ^30^ Department of Urology, School of Medicine, College of Medicine, Taipei Medical University, Taipei, Taiwan; ^31^ Department of Urology, Taipei Medical University Hospital, Taipei Medical University, Taipei, Taiwan; ^32^ Division of Urology, Department of Surgery, Chang Gung Memorial Hospital, Chiayi, Taiwan; ^33^ Chang Gung University of Science and Technology, Chiayi, Taiwan; ^34^ Department of Medicine, College of Medicine, Chang Gung University, Taoyuan, Taiwan; ^35^ Department of Urology, Shuang Ho Hospital, Taipei Medical University, New Taipei City, Taiwan; ^36^ Taipei Medical University Research Center of Urology and Kidney (TMU-RCUK), Taipei Medical University, Taipei, Taiwan; ^37^ Division of Urology, Department of Surgery, Chang Gung Memorial Hospital, Taoyuan, Taiwan; ^38^ Graduate Institute of Clinical Medical Science, College of Medicine, Chang Gung University, Taoyuan, Taiwan; ^39^ School of Medicine, College of Medicine, Chang Gung University, Taoyuan, Taiwan; ^40^ School of Medicine, College of Medicine, Kaohsiung Medical University, Kaohsiung, Taiwan; ^41^ Cohort Research Center, Kaohsiung Medical University, Kaohsiung, Taiwan

**Keywords:** hematuria, flank pain, symptomatic hydronephrosis, upper tract urothelial carcinoma (UTUC), radical nephroureterectomy (RNU), prognosis

## Abstract

**Purpose:**

We aimed to evaluate the impact of preoperative local symptoms on prognosis after radical nephroureterectomy in patients with upper tract urothelial carcinoma (UTUC).

**Methods:**

This retrospective study consisted of 2,662 UTUC patients treated at 15 institutions in Taiwan from 1988 to 2019. Clinicopathological data were retrospectively collected for analysis by the Taiwan UTUC Collaboration Group. The Kaplan-Meier method was used to calculate overall survival (OS), cancer-specific survival (CSS), disease-free survival (DFS), and bladder recurrence-free survival (BRFS). The prognostic value of preoperative local symptoms in OS, CSS, DFS, and BRFS was investigated using Cox proportional hazards models.

**Results:**

The median follow-up was 36.6 months. Among 2,662 patients, 2,130 (80.0%) presented with hematuria and 398 (15.0%) had symptomatic hydronephrosis at diagnosis. Hematuria was associated with less symptomatic hydronephrosis (*p <*0.001), more dialysis status (*p* = 0.027), renal pelvic tumors (*p <*0.001), and early pathological tumor stage (*p* = 0.001). Symptomatic hydronephrosis was associated with female patients (*p <*0.001), less dialysis status (*p* = 0.001), less bladder cancer history (*p <*0.001), ureteral tumors (*p <*0.001), open surgery (*p* = 0.006), advanced pathological tumor stage (*p <*0.001), and postoperative chemotherapy (*p* = 0.029). Kaplan-Meier analysis showed that patients with hematuria or without symptomatic hydronephrosis had significantly higher rates of OS, CSS, and DFS (all *p <*0.001). Multivariate analysis confirmed that presence of hematuria was independently associated with better OS (HR 0.789, 95% CI 0.661–0.942) and CSS (HR 0.772, 95% CI 0.607–0.980), while symptomatic hydronephrosis was a significant prognostic factor for poorer OS (HR 1.387, 95% CI 1.142–1.683), CSS (HR 1.587, 95% CI 1.229–2.050), and DFS (HR 1.378, 95% CI 1.122–1.693).

**Conclusions:**

Preoperative local symptoms were significantly associated with oncological outcomes, whereas symptomatic hydronephrosis and hematuria had opposite prognostic effects. Preoperative symptoms may provide additional information on risk stratification and perioperative treatment selection for patients with UTUC.

## Introduction

Urothelial carcinoma (UC) is a malignant tumor of the lining of the urinary tract. While the majority of UCs arise in the bladder, upper tract UC (UTUC) is less common (1, 2). Unlike bladder UC, almost 60% of UTUCs are invasive at diagnosis (3–5). The standard treatment for invasive UTUC is radical nephroureterectomy (RNU) with bladder cuff excision, but the cancer often recurs after surgical intervention (2, 3). Therefore, it is important to select candidates who require adjunctive therapy in the perioperative period. Identifying useful prognostic markers for UTUC is one way to aid patient selection. Previous studies have demonstrated the prognostic significance of pathological features such as tumor stage and lymphovascular invasion (2). However, these characteristics are only obtained after RNU and cannot be incorporated into preoperative assessment. Preoperative prognostic factors are more conducive to make up for the inadequacy of clinical staging of UTUC.

UTUC is usually initially diagnosed by examination after seeking medical attention for clinical symptoms. The most common symptom of UTUC is hematuria, which occurs in approximately 70%–80% of patients (2, 6). Flank pain is the second most common symptom (20%), usually caused by obstruction of urine flow, and is closely related to hydronephrosis (7–9). Other systemic symptoms, including weight loss, general malaise, fatigue, and cachexia, may be associated with worse prognosis and should be treated promptly (2, 8, 9). Although local symptoms are common, their impact on survival outcomes in patients with UTUC remains questionable (7–14). This study aimed to evaluate the value of preoperative local symptoms on the prognosis of UTUC after nephroureterectomy.

## Materials and Methods

### Patient Collection and Tumor Specimens

The Taiwan UTUC Collaboration Group collected 4,813 patients from 15 institutions in Taiwan from 1988 to 2019. A total of 2,662 patients were included in this study after we excluded patients who underwent surgery other than RNU or who had incomplete medical records. This study was supervised by the review board of our institution (KMUHIRB-E(I)-20180214). RNU was performed with either an open or laparoscopic approach. The open approach used one or two incisions, such as a midline incision or a flank plus Gibson incision, while the laparoscopic approach employed a camera port to minimize trauma caused by the incision through a transperitoneal or retroperitoneal access. Lymph node dissection was performed when lymph node involvement was suspected on preoperative imaging or when lymphadenopathy was found during surgery. Intravesical chemotherapy was not routinely performed after RNU, except in patients with bladder recurrence during follow-up. Various clinicopathological data were included for analysis, including age, gender, smoking, local symptoms, dialysis, bladder cancer history, tumor location, surgical approach, pathological features (pathological T stage, pathological N stage, tumor grade, multifocality), and postoperative chemotherapy.

All tumor specimens were reviewed by genitourinary pathologists at each medical center, and the criteria for pathological characteristics were uniform. Tumor stage was defined according to the 2010 American Joint Committee Cancer TNM (Tumor, Lymph Node, Metastasis) system (15), while tumor grade was based on the 2004 World Health Organization/International Society of Urologic Pathology Consensus Classification (16).

### Preoperative Symptom Assessment

Patients with hematuria may or may not have visible red urine. The presence of hematuria was determined by urinalysis prior to the diagnosis of UTUC. Patients were considered to have hematuria if urinalysis revealed more than 3 red blood cells per high-power field on two consecutive microscopic evaluations. We used renal ultrasonography, computed tomography, magnetic resonance imaging, or intravenous pyelography to detect hydronephrosis. Consistent with previous studies (10, 17), any degree of dilatation of the renal collecting system was defined as hydronephrosis. If the pelvicalyceal dilatation was caused by noncancerous condition such as urolithiasis and benign ureteral stricture, it was not considered hydronephrosis. Symptomatic hydronephrosis was defined as moderate to severe flank pain on the same side of the hydronephrosis (10). Patients with nonspecific lumbago or flank pain contralateral to UTUC were not considered symptomatic hydronephrosis. Patients were divided into two groups according to the presence of hematuria or symptomatic hydronephrosis to assess the prognostic value of local symptoms.

### Follow-Up

Typically, patients were followed up every 3 months for the first 2 years after RNU. Follow-up visits were performed every 6 months from years 3 to 4, and annually after year 5 if there was no disease recurrence. Workup included a thorough history taking, physical examination, urine cytology, blood tests, chest X-ray, cystoscopy, and abdominal computed tomography. Disease progression was defined as distant metastasis or cancer development in the tumor bed or regional lymph nodes. Bladder recurrence was considered an independent entity for survival analysis. Cancer-specific and overall mortality was determined by reviewing death certificates and medical records.

### Statistical Analysis

Differences in categorical parameters between the presence and absence of each local symptom were assessed by Pearson’s chi-square test, and continuous variables were compared by Student’s *t* test. The Kaplan-Meier method was used to evaluate the effect of local symptoms on overall survival (OS), cancer-specific survival (CSS), disease-free survival (DFS), and bladder recurrence-free survival (BRFS). Survival curves were compared by log-rank test, and survival time from surgery date to each endpoint (i.e., all-cause death, cancer-specific mortality, disease progression, bladder recurrence) or last visit was calculated. In addition, we used Cox proportional hazards models to assess the effect of each variable on oncological outcomes. The effects of all variables on each survival rate were examined in univariate analysis, and statistically significant variables were adjusted to evaluate their prognostic value in multivariate analysis. We used SPSS 26.0 (SPSS Inc., Chicago, IL, USA) for all analyses and *p <*0.05 was defined as statistically significant.

## Results

### Clinicopathological Data and Preoperative Symptoms

The median and mean follow-up was 36.6 and 47.7 months, respectively. This study consisted of 2,662 UTUC patients, including 1,465 (55.0%) women and 1,197 (45.0%) men. Demographic and clinicopathological characteristics were compared according to the presence of hematuria (**Table 1**) and symptomatic hydronephrosis (**Table 2**). Hematuria occurred in 2,130 (80.0%) patients. **Table 1** shows that patients with hematuria were associated with less symptomatic hydronephrosis (*p <*0.001), more dialysis status (*p* = 0.027), renal pelvic tumors (*p <*0.001), early pathological T stage (*p* = 0.001), less disease progression (*p* = 0.007), fewer cancer-specific deaths (*p <*0.001), and fewer all-cause deaths (*p* = 0.001). Several parameters were found to be not significantly different between the two groups, including age (*p* = 0.186), gender (*p* = 0.905), smoking (*p* = 0.443), bladder cancer history (*p* = 0.671), surgical approach (*p* = 0.732), pathological N stage (*p* = 0.789), tumor grade (*p* = 0.189), multifocality (*p* = 0.611), postoperative chemotherapy (*p* = 0.273), and bladder recurrence (*p* = 0.886).

**Table 1:** Clinicopathological data of UTUC patients according to hematuria.

Variables	No Hematuria (n = 532)		Hematuria (n = 2130)		*p* value^a^
	N	%	N	%	
Age (mean/SD)^b^	68.8	(10.8)	69.0	(10.9)	0.186
Gender					0.905
Female	294	(55.3)	1171	(55.0)	
Male	238	(44.7)	959	(45.0)	
Smoking					0.443
No	401	(75.4)	1639	(76.9)	
Yes	131	(24.6)	491	(23.1)	
Symptomatic hydronephrosis					<0.001**
No	368	(69.2)	1896	(89.0)	
Yes	164	(30.8)	234	(11.0)	
Dialysis					0.027*
No	470	(88.3)	1801	(84.6)	
Yes	62	(11.7)	329	(15.4)	
Bladder cancer history					0.671
No	412	(77.4)	1631	(76.6)	
Yes	120	(22.6)	499	(23.4)	
Tumor location					<0.001**
Renal pelvis	179	(33.6)	1051	(49.3)	
Ureter	249	(46.8)	671	(31.5)	
Synchronous	104	(19.5)	408	(19.2)	
Surgical approach					0.732
Open	180	(33.8)	704	(33.1)	
Laparoscopy	352	(66.2)	1426	(66.9)	
Pathological T stage					0.001**
pTis/pTa	79	(14.8)	366	(17.2)	
pT1	110	(20.7)	578	(27.1)	
pT2	106	(19.9)	423	(19.9)	
pT3	1097	(37.0)	659	(30.9)	
pT4	40	(7.5)	104	(4.9)	
Pathological N stage					0.789
pN0	131	(24.6)	554	(26.0)	
pNx	368	(69.2)	1441	(67.7)	
pN+	33	(6.2)	135	(6.3)	
Tumor grade					0.189
Low grade	75	(14.1)	350	(16.4)	
High grade	457	(85.9)	1780	(83.6)	
Multifocality					0.611
No	352	(66.2)	1434	(67.3)	
Yes	180	(33.8)	696	(32.7)	
Postoperative chemotherapy					0.273
No	434	(81.6)	1780	(83.6)	
Yes	98	(18.4)	350	(16.4)	
Bladder recurrence					0.886
No	388	(72.9)	1560	(73.2)	
Yes	144	(27.1)	570	(26.8)	
Disease progression					0.007**
No	378	(71.1)	1634	(76.7)	
Yes	154	(28.9)	496	(23.3)	
Cancer-specific death					<0.001**
No	430	(80.8)	1854	(87.0)	
Yes	102	(19.2)	276	(13.0)	
All-cause death					0.001**
No	352	(66.2)	1563	(73.4)	
Yes	180	(33.8)	567	(26.6)	

^a^Chi-square test calculated for the difference in variables. ^b^Student’s t test calculated for the difference in means. * < 0.05, ** < 0.01.

**Table 2:** Clinicopathological data of UTUC patients according to symptomatic hydronephrosis.

Variables	No Symptomatic hydronephrosis (n = 2264)		Symptomatic hydronephrosis (n = 398)		*p* value^a^
	N	%	N	%	
Age (mean/SD)^b^	68.9	(11.1)	69.0	(10.1)	0.864
Gender					<0.001**
Female	1214	(53.6)	251	(63.1)	
Male	1050	(46.4)	147	(36.9)	
Smoking					0.072
No	1721	(76.0)	319	(80.2)	
Yes	543	(24.0)	79	(19.8)	
Hematuria					<0.001**
No	368	(16.3)	164	(41.2)	
Yes	1896	(83.7)	234	(58.8)	
Dialysis					0.001**
No	1909	(84.3)	362	(91.0)	
Yes	355	(15.7)	36	(9.0)	
Bladder cancer history					<0.001**
No	1707	(75.4)	336	(84.4)	
Yes	557	(24.6)	62	(15.6)	
Tumor location					<0.001**
Renal pelvis	1104	(48.8)	126	(31.7)	
Ureter	731	(32.3)	189	(47.5)	
Synchronous	429	(18.9)	83	(20.9)	
Surgical approach					0.006**
Open	728	(32.2)	156	(39.2)	
Laparoscopy	1536	(67.8)	242	(60.8)	
Pathological T stage					<0.001**
pTis/pTa	384	(17.0)	61	(15.3)	
pT1	619	(27.3)	69	(17.3)	
pT2	434	(19.2)	95	(23.9)	
pT3	715	(31.6)	141	(35.4)	
pT4	112	(4.9)	32	(8.0)	
Pathological N stage					0.140
pN0	592	(26.1)	93	(23.4)	
pNx	1537	(67.9)	272	(68.3)	
pN+	135	(6.0)	33	(8.3)	
Tumor grade					0.157
Low grade	371	(16.4)	54	(13.6)	
High grade	1893	(83.6)	344	(86.4)	
Multifocality					0.815
No	1521	(67.2)	265	(66.6)	
Yes	743	(32.8)	133	(33.4)	
Postoperative chemotherapy					0.029*
No	1898	(83.8)	316	(79.4)	
Yes	366	(16.2)	82	(20.6)	
Bladder recurrence					0.015*
No	1637	(72.3)	311	(78.1)	
Yes	627	(27.7)	87	(21.9)	
Disease progression					<0.001**
No	1741	(76.9)	271	(68.1)	
Yes	523	(23.1)	127	(31.9)	
Cancer-specific death					<0.001**
No	1974	(87.2)	310	(77.9)	
Yes	290	(12.8)	88	(22.1)	
All-cause death					<0.001**
No	1660	(73.3)	255	(64.1)	
Yes	604	(26.7)	143	(35.9)	

^a^Chi-square test calculated for the difference in variables. ^b^Student’s t test calculated for the difference in means. * < 0.05, ** < 0.01.

In **Table 2**, 398 (15.0%) patients had symptomatic hydronephrosis at the initial presentation. They were associated with female patients (*p <*0.001), less hematuria (*p <*0.001), less dialysis status (*p* = 0.001), less bladder cancer history (*p <*0.001), ureteral tumors (*p <*0.001), open surgical approach (*p* = 0.006), advanced pathological T stage (*p <*0.001), postoperative chemotherapy (*p* = 0.029), less bladder recurrence (*p* = 0.015), more disease progression (*p <*0.001), more cancer-specific mortality (*p <*0.001), and more all-cause mortality (*p <*0.001). No differences in age (*p* = 0.864), smoking (*p* = 0.072), pathological N stage (*p* = 0.140), tumor grade (*p* = 0.157), and multifocality (*p* = 0.815) were observed between the two groups.

### Kaplan-Meier Analysis of OS, CSS, DFS, and BRFS Based on Hematuria or Symptomatic Hydronephrosis

**Figure 1:**
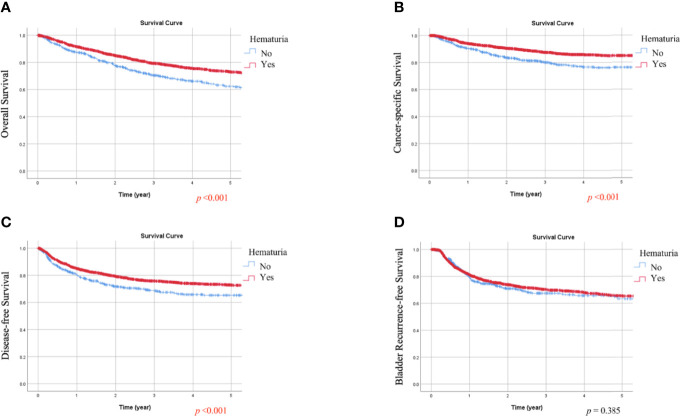
Kaplan-Meier estimates of overall survival **(A)**, cancer-specific survival **(B)**, disease-free survival **(C)**, and bladder recurrence-free survival **(D)** according to hematuria.

**Figure 2:**
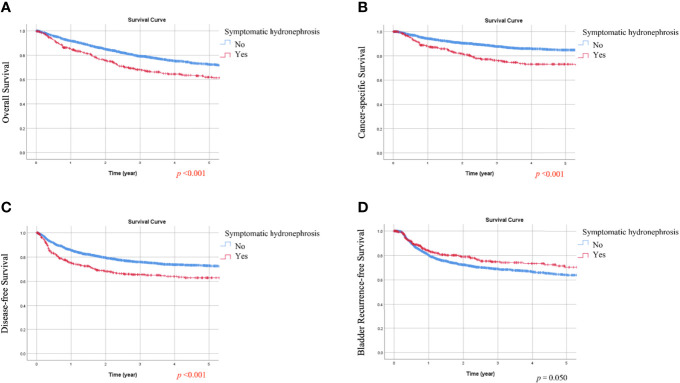
Kaplan-Meier estimates of the overall survival **(A)**, cancer-specific survival **(B)**, disease-free survival **(C)**, and bladder recurrence-free survival **(D)** according to symptomatic hydronephrosis.

During follow-up, all-cause death, cancer-specific mortality, disease progression, and bladder recurrence occurred in 747 (28.1%), 378 (14.2%), 650 (24.4%), and 714 (26.8%) patients, respectively. As shown in **Tables 1** and **2**, the absence of hematuria or the presence of symptomatic hydronephrosis was significantly associated with more crude events in OS, CSS, and DFS (all *p <*0.01). In Kaplan-Meier analysis, patients with hematuria had significantly better OS, CSS, and DFS than those without hematuria (**Figures 1A–C**, respectively; all *p <*0.001). In contrast, patients with symptomatic hydronephrosis had significantly lower OS, CSS, and DFS than cases without symptomatic hydronephrosis (**Figures 2A–C**, respectively; all *p <*0.001). For BRFS, there was no significant difference according to hematuria (**Figure 1D**; *p* = 0.385) or symptomatic hydronephrosis (**Figure 2D**; *p* = 0.050).

### Cox Proportional Hazards Models for OS, CSS, DFS, and BRFS

In univariate analysis (**Table 3**), multiple variables were associated with poorer OS, CSS, and DFS, including advanced age, synchronous renal pelvic and ureteral tumors, open surgical approach, advanced pathological T stage, lymph node metastasis, high tumor grade, multifocality, postoperative chemotherapy, absence of hematuria, and symptomatic hydronephrosis. As for bladder recurrence, gender, smoking, bladder cancer history, tumor location, pathological T stage, and multifocality were significantly associated with BRFS.

**Table 3:** Comparative univariate survival analysis of UTUC patients.

Univariate analysis		OS		CSS		DFS		BRFS	
		HR (95% CI)	*p* value	HR (95% CI)	*p* value	HR (95% CI)	*p* value	HR (95% CI)	*p* value
Age		1.036 (1.028, 1.043)	<0.001**	1.023 (1.013, 1.033)	<0.001**	1.013 (1.006, 1.021)	0.001**	0.998 (0.991, 1.005)	0.621
Gender			0.241		0.048*		0.021*		<0.001**
Female		1		1		1		1	
Male		1.090 (0.944, 1.258)		1.225 (1.002, 1.499)		1.199 (1.028, 1.399)		1.762 (1.519, 2.043)	
Smoking			0.396		0.278		0.069		<0.001**
No		1		1		1		1	
Yes		1.074 (0.911, 1.267)		1.135 (0.903, 1.428)		1.175 (0.987, 1.399)		1.401 (1.191, 1.647)	
Dialysis			0.387		<0.001**		0.003**		0.292
No		1		1		1		1	
Yes		1.094 (0.893, 1.339)		0.496 (0.337, 0.729)		0.675 (0.522, 0.874)		1.116 (0.910, 1.370)	
Bladder cancer history			0.001**		0.126		0.004**		<0.001**
No	1			1		1		1	
Yes	1.310 (1.112, 1.543)			1.199 (0.950, 1.513)		1.288 (1.082, 1.533)		1.818 (1.552, 2.129)	
Tumor location			<0.001**		<0.001**		<0.001**		<0.001**
Renal pelvis	1			1		1		1	
Ureter	1.155 (0.978, 1.365)		0.089	1.036 (0.815, 1.316)	0.775	1.006 (0.839, 1.207)	0.946	1.374 (1.164, 1.622)	<0.001**
Synchronous	1.746 (1.456, 2.094)		<0.001**	1.879 (1.467, 2.406)	<0.001**	1.790 (1.480, 2.165)	<0.001**	1.598 (1.314, 1.945)	<0.001**
Surgical approach			<0.001**		<0.001**		0.001**		0.784
Open	1			1		1		1	
Laparoscopy	0.715 (0.618, 0.828)			0.630 (0.514, 0.773)		0.769 (0.656, 0.900)		1.022 (0.874, 1.195)	
Pathological T stage			<0.001**		<0.001**		<0.001**		0.005**
pTis/pTa	1			1		1		1	
pT1	1.248 (0.937, 1.662)		0.130	1.560 (0.771, 3.157)	0.216	1.299 (0.858, 1.967)	0.216	1.256 (1.002, 1.575)	0.048*
pT2	1.759 (1.321, 2.342)		<0.001**	5.034 (2.649, 9.568)	<0.001**	3.329 (2.275, 4.873)	<0.001**	1.272 (1.003, 1.614)	0.047*
pT3	3.417 (2.642, 4.419)		<0.001**	13.738 (7.489, 25.199)	<0.001**	7.312 (5.136, 10.410)	<0.001**	1.218 (0.972, 1.526)	0.087
pT4	9.955 (7.279, 13.61)		<0.001**	41.709 (21.983, 79.14)	<0.001**	19.294 (13.004, 28.63)	<0.001**	0.435 (0.228, 0.830)	0.012*
Pathological N stage			<0.001**		<0.001**		<0.001**		0.468
pN0	1			1		1		1	
pNx	1.043 (0.875, 1.244)		0.638	0.955 (0.741, 1.231)	0.722	0.999 (0.825, 1.208)	0.988	1.042 (0.880, 1.235)	0.631
pN+	4.742 (3.673, 6.123)		<0.001**	6.501 (4.744, 8.908)	<0.001**	5.677 (4.432, 7.273)	<0.001**	0.822 (0.544, 1.240)	0.349
Tumor grade			<0.001**		<0.001**		<0.001**		0.654
Low grade	1			1		1		1	
High grade	2.317 (1.830, 2.933)			6.833 (3.927, 11.891)		3.666 (2.670, 5.032)		0.959 (0.797, 1.153)	
Multifocality			<0.001**		<0.001**		<0.001**		<0.001**
No	1			1		1		1	
Yes	1.576 (1.361, 1.824)			1.860 (1.518, 2.278)		1.807 (1.547, 2.110)		1.335 (1.145, 1.556)	
Postoperative chemotherapy			<0.001**		<0.001**		<0.001**		0.598
No	1			1		1		1	
Yes	1.646 (1.387, 1.954)			2.361 (1.899, 2.937)		2.521 (2.135, 2.977)		1.053 (0.868, 1.278)	
Hematuria			<0.001**		<0.001**		0.002**		0.385
No	1			1		1		1	
Yes	0.707 (0.598, 0.836)			0.626 (0.499, 0.785)		0.744 (0.621, 0.891)		0.922 (0.768, 1.107)	
Symptomatic hydronephrosis		<0.001**		<0.001**		<0.001**		0.051
No	1			1		1		1	
Yes	1.485 (1.237, 1.782)			1.883 (1.483, 2.390)		1.514 (1.247, 1.838)		0.800 (0.639, 1.001)	

CI, confidence interval; HR, hazard ratio; OS, overall survival; CSS, cancer-specific survival; DFS, disease-free survival; BRFS, bladder recurrence-free survival; * < 0.05, ** < 0.01.

After adjusting for significant variables in multivariate analysis (**Table 4**), bladder cancer history was associated with worse OS and DFS. Statistically significant factors for worse OS and CSS were open surgical approach and absence of hematuria. Variables independently associated with worse OS, CSS, and DFS were advanced age, advanced pathological T stage, lymph node metastasis, high tumor grade, and symptomatic hydronephrosis. In multivariate analysis of BRFS, gender, tumor location, bladder cancer history, and pathological T stage were significantly associated with bladder recurrence.

**Table 4:** Comparative multivariate survival analysis of UTUC patients.

Multivariate analysis	OS		CSS		DFS		BRFS	
	HR (95% CI)	*p* value	HR (95% CI)	*p* value	HR (95% CI)	*p* value	HR (95% CI)	*p* value
Age	1.039 (1.031, 1.047)	<0.001**	1.020 (1.010, 1.031)	<0.001**	1.011 (1.003, 1.019)	0.009**	1.001 (0.994, 1.008)	0.825
Gender		0.165		0.097		0.177		<0.001**
Female	1		1		1		1	
Male	1.128 (0.952, 1.337)		1.223 (0.964, 1.551)		1.134 (0.945, 1.361)		1.685 (1.421, 1.997)	
Smoking		0.180		0.663		0.370		0.966
No	1		1		1		1	
Yes	1.143 (0.940, 1.390)		1.062 (0.811, 1.389)		1.099 (0.894, 1.350)		0.996 (0.826, 1.202)	
Dialysis		<0.001**		0.137		0.553		0.619
No	1		1		1		1	
Yes	1.592 (1.277, 1.984)		0.735 (0.490, 1.103)		0.920 (0.699, 1.211)		1.057 (0.848, 1.318)	
Bladder cancer history		0.035*		0.100		0.020*		<0.001**
No	1		1		1		1	
Yes	1.206 (1.013, 1.436)		1.232 (0.961, 1.580)		1.248 (1.036, 1.503)		1.616 (1.366, 1.912)	
Tumor location		0.103		0.631		0.702		0.001**
Renal pelvis	1		1		1		1	
Ureter	1.176 (0.985, 1.404)	0.073	1.121 (0.868, 1.447)	0.382	1.053 (0.869, 1.277)	0.597	1.335 (1.122, 1.588)	0.001**
Synchronous	1.222 (0.959, 1.557)	0.104	1.108 (0.803, 1.529)	0.533	1.105 (0.861, 1.418)	0.434	1.417 (1.097, 1.831)	0.008**
Surgical approach		0.003**		0.022*		0.533		0.612
Open	1		1		1		1	
Laparoscopy	0.796 (0.685, 0.925)		0.783 (0.634, 0.966)		0.950 (0.807, 1.117)		1.043 (0.887, 1.225)	
Pathological T stage		<0.001**		<0.001**		<0.001**		0.010*
pTis/pTa	1		1		1		1	
pT1	1.200 (0.894, 1.609)	0.224	1.283 (0.628, 2.620)	0.494	1.156 (0.758, 1.763)	0.502	1.286 (1.018, 1.626)	0.035*
pT2	1.443 (1.061, 1.961)	0.019*	3.328 (1.713, 6.464)	<0.001**	2.563 (1.717, 3.824)	<0.001**	1.225 (0.944, 1.590)	0.127
pT3	2.802 (2.102, 3.734)	<0.001**	8.618 (4.561, 16.281)	<0.001**	5.062 (3.456, 7.413)	<0.001**	1.213 (0.933, 1.576)	0.149
pT4	6.124 (4.282, 8.759)	<0.001**	17.812 (8.949, 35.453)	<0.001**	9.731 (6.280, 15.079)	<0.001**	0.466 (0.238, 0.912)	0.026*
Pathological N stage		<0.001**		<0.001**		<0.001**		0.635
pN0	1		1		1		1	
pNx	1.145 (0.957, 1.371)	0.139	1.124 (0.869, 1.455)	0.374	1.136 (0.936, 1.379)	0.198	1.081 (0.908, 1.286)	0.380
pN+	2.670 (2.028, 3.516)	<0.001**	2.841 (2.029, 3.979)	<0.001**	2.611 (1.998, 3.413)	<0.001**	0.977 (0.637, 1.498)	0.915
Tumor grade		0.008**		0.002**		0.003**		0.449
Low grade	1		1		1		1	
High grade	1.419 (1.095, 1.839)		2.512 (1.406, 4.487)		1.656 (1.181, 2.324)		0.924 (0.752, 1.134)	
Multifocality		0.237		0.091		0.009**		0.608
No	1		1		1		1	
Yes	1.135 (0.920, 1.399)		1.283 (0.961, 1.714)		1.343 (1.077, 1.674)		1.057 (0.855, 1.306)	
Postoperative chemotherapy		0.857		0.499		0.284		0.578
No	1		1		1		1	
Yes	1.018 (0.839, 1.234)		0.920 (0.723, 1.171)		1.108 (0.919, 1.336)		1.065 (0.854, 1.328)	
Hematuria		0.009**		0.034*		0.162		0.455
No	1		1		1		1	
Yes	0.789 (0.661, 0.942)		0.772 (0.607, 0.980)		0.874 (0.723, 1.056)		0.930 (0.769, 1.125)	
Symptomatic hydronephrosis		0.001**		<0.001**		0.002**		0.136
No	1		1		1		1	
Yes	1.387 (1.142, 1.683)		1.587 (1.229, 2.050)		1.378 (1.122, 1.693)		0.837 (0.662, 1.057)	

CI, confidence interval; HR, hazard ratio; OS, overall survival; CSS, cancer-specific survival; DFS, disease-free survival; BRFS, bladder recurrence-free survival; * < 0.05, ** < 0.01.

In summary, the presence of hematuria was associated with better OS and CSS in univariate analysis (HR 0.707, 95% CI 0.598–0.836 and HR 0.626, 95% CI 0.499–0.785) and multivariate analysis (HR 0.789, 95% CI 0.661–0.942 and HR 0.772, 95% CI 0.607–0.980). On the other hand, symptomatic hydronephrosis was a significant prognostic factor for worse OS, CSS, and DFS in univariate analysis (HR 1.485, 95% CI 1.237–1.782, HR 1.883, 95% CI 1.483–2.390, and HR 1.514, 95% CI 1.247–1.838, respectively) and multivariate analysis (HR 1.387, 95% CI 1.142–1.683, HR 1.587, 95% CI 1.229–2.050, and HR 1.378, 95% CI 1.122–1.693, respectively).

## Discussion

Studies have shown that systemic symptoms such as weight loss, general malaise, and fatigue are associated with poor prognosis in UTUC (2, 8, 9). However, the prognostic significance of local symptoms directly attributable to the tumor, such as hematuria and symptomatic hydronephrosis, have been poorly studied. This is the largest multicenter study investigating the prognostic value of preoperative local symptoms in UTUC, showing that hematuria and symptomatic hydronephrosis independently lead to better and worse survival, respectively. These findings suggest that hematuria and symptomatic hydronephrosis are not only helpful in disease detection, but also have prognostic value.

Although hematuria is the most common symptom of UC, few studies have investigated the prognostic role of hematuria. Ramirez et al. showed that the severity of hematuria was associated with more advanced pathological stages of bladder UC (18). However, inherent anatomical differences between the bladder and upper urinary tract may prevent extrapolation of this result to UTUC (4). Bladder UC is almost impossible to block the urinary tract before hematuria occurs. In contrast, UTUC is prone to urinary obstruction due to the small diameter of the upper urinary tract and may not present with hematuria. In the absence of hematuria, accurate diagnosis may be delayed, leading to tumor upstaging. As our results showed, the pathological tumor stage was significantly higher in patients without hematuria than in patients with hematuria (*p* = 0.001).

Previous studies have not established the protective effect of hematuria on UTUC (10–13, 19). Of these studies, some of them showed that hematuria was associated with better prognosis in patients with UTUC (12, 13), but not others (10, 11, 19). In the present study, the significance of hematuria in OS and CSS remained after adjustment for various clinicopathological variables. With three times the number of patients compared to the largest previous study (11), we believe that patients presenting with hematuria have better survival rates. Notably, the results were very similar if only gross hematuria was defined as hematuria (**Supplementary Table**).

Another key finding of the study was that symptomatic hydronephrosis predicted worse outcomes. Similar to the results of hematuria, not all previous studies showed that flank pain (11–13) or hydronephrosis (20–24) was unfavorable for the prognosis of UTUC. In current guidelines and in a recent meta-analysis, hydronephrosis is considered a high-risk feature (2, 25), but its prognostic significance is greatly reduced after multivariate adjustment (20, 21). Although high-grade hydronephrosis may be a better indicator of poor prognosis (22–24), interobserver variability and how severe hydronephrosis is significant are problematic. Symptomatic hydronephrosis was described as co-occurring flank pain and hydronephrosis, which was clearly defined and confirmed as an important prognostic factor for UTUC in a previous study (10). Notably, neither flank pain nor hydronephrosis alone was significant in multivariate analysis. Our results also supported the prognostic value of symptomatic hydronephrosis in UTUC.

Some previous studies grouped all local symptoms as a whole to assess their impact on prognosis. For example, in patients with renal cell carcinoma (RCC), those with flank pain, hematuria, and palpable tumors had a worse prognosis than those without these symptoms (26–28). Raman et al. used the same criteria, but local symptoms failed to predict outcomes in patients with UTUC (8). Although the etiology of flank pain can differ between RCC and UTUC (local mass effect and urinary tract obstruction, respectively), pain is probably related to tumor aggressiveness. On the contrary, hematuria generally represents the invasion of advanced RCC into the collecting system, but is a warning symptom for early diagnosis and prompt treatment of UTUC. In addition, Ataus et al. showed that in UTUC, patients with flank pain had lower survival than those with hematuria (13). Taken together, we believe that dissecting local symptoms in detail is important to obtain additional prognostic information.

Zhao et al. found that UTUC patients with hydronephrosis should have a shorter waiting time for surgery (13), otherwise the increased intraluminal pressure may lead to wall thinning and a greater chance of peripheral invasion of tumor cells (29). Since symptomatic hydronephrosis was a more reliable predictor of cancer invasiveness than hydronephrosis alone (10), we supposed that these patients may require more timely treatment to avoid upstaging. Another potential clinical application is for monitoring treatment efficacy. In the study by Miyake et al., down-grading of hydronephrosis after neoadjuvant chemotherapy was associated with favorable oncological outcomes (30). Likewise, relief of flank pain could reflect a favorable response to therapy and a proxy for downstaging.

This study has some limitations. First, this is a retrospective study. Second, we were unable to determine the exact extent of the patient’s local symptoms. Third, several important factors, such as surgical margins, histological variants, and tumor necrosis, were not included in the analysis because information on many patients was not available. We performed a sensitivity analysis in a limited subgroup and found similar results. To provide more information to those who may be wondering, we analyzed the effect of positive surgical margins on survival at different pT stages. As shown in the **Supplementary Figure**, positive surgical margins appeared to have the greatest impact on pT3 disease. Fourth, data collection was performed by collaborating with multiple medical centers, so surgical specimens were reviewed by different genitourinary pathologists and operated by different surgeons. To our knowledge, there is no consensus on the impact of local symptoms on the prognosis of UTUC. We have included most of the recognized clinicopathological variables in our comprehensive survival analysis, and this is the largest multicenter study to date evaluating the effect of preoperative symptoms on UTUC outcomes. We demonstrate that preoperative local symptoms are important prognostic factors, and our promising results support further prospective studies for validation.

In conclusion, symptomatic hydronephrosis was an independent prognostic factor for worse disease outcomes, while the presence of hematuria was associated with better survival. Preoperative local symptoms could be a novel variable to risk stratify patients with UTUC and help physicians make better treatment decisions.

## Data Availability Statement

The original contributions presented in the study are included in the article/**Supplementary Material**. Further inquiries can be directed to the corresponding authors.

## Ethics Statement

The studies involving human participants were reviewed and approved by Kaohsiung Medical University Hospital [KMUHIRB-E(I)-20180214]. The patients/participants provided their written informed consent to participate in this study.

## Author Contributions

H-CY and W-JW conceived the project. All authors collected the data. H-CY analyzed the results. T-WL and H-CY drafted the manuscript. H-CY and W-JW edited the manuscript. All authors contributed to the article and approved the submitted version.

## Funding

This study was supported by Kaohsiung Municipal Ta-Tung Hospital (kmtth-110-015) and supported partially by the Ministry of Science and Technology (MOST 109-2314-B-037-095), Ministry of Health and Welfare (MOHW111-TDU-B-212-134006), Kaohsiung Medical University Hospital (KMUH-DK-111007C), Kaohsiung Medical University Regenerative Medicine and Cell Therapy Research Center (KMU-TC109A02), and Kaohsiung Medical University Center for Liquid Biopsy and Cohort Research (KMU-TC109B05).

## Conflict of Interest

The authors declare that the research was conducted in the absence of any commercial or financial relationships that could be construed as a potential conflict of interest.

## Publisher’s Note

All claims expressed in this article are solely those of the authors and do not necessarily represent those of their affiliated organizations, or those of the publisher, the editors and the reviewers. Any product that may be evaluated in this article, or claim that may be made by its manufacturer, is not guaranteed or endorsed by the publisher.

## Supplementary Material

Supplementary Figure Kaplan-Meier analyses of overall survival, cancer-specific survival, disease-free survival, and bladder recurrence-free survival based on positive surgical margins and stratified for different pT stages.
