# Supplementary figures and images for: The Value of Preoperative Local Symptoms in Prognosis of Upper Tract Urothelial Carcinoma After Radical Nephroureterectomy: A Retrospective, Multicenter Cohort Study
Source: Front Oncol. 2022 Jun 2;12:872849. doi: 10.3389/fonc.2022.872849 (PMC9201473; doi:10.3389/fonc.2022.872849)

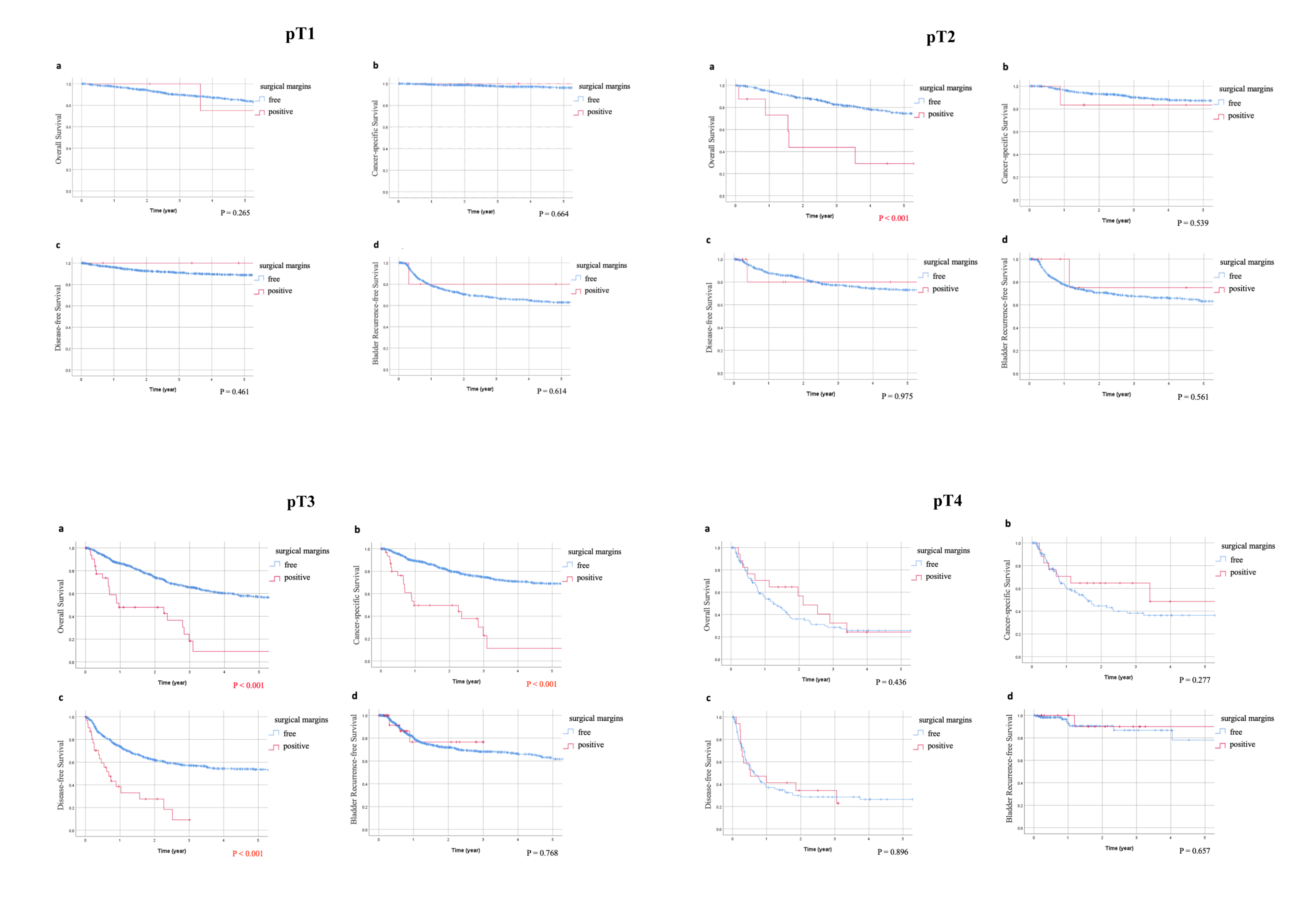

Supplement: Supplementary Figure — Kaplan-Meier analyses of overall survival, cancer-specific survival, disease-free survival, and bladder recurrence-free survival based on positive surgical margins and stratified for different pT stages. [file Image_1.tiff]
