# Supplementary material for: The Value of Preoperative Local Symptoms in Prognosis of Upper Tract Urothelial Carcinoma After Radical Nephroureterectomy: A Retrospective, Multicenter Cohort Study
Source: Front Oncol. 2022 Jun 2;12:872849. doi: 10.3389/fonc.2022.872849 (PMC9201473; doi:10.3389/fonc.2022.872849)
Supplement: Supplementary file 2 [file Table_1.pdf]

SUPPLEMENTARY TABLE Comparative multivariate survival analysis of UTUC patients

Multivariate analysis	OS		CSS		DFS		BRFS	
	HR (95% CI)	*p* value	HR (95% CI)	*p* value	HR (95% CI)	*p* value	HR (95% CI)	*p* value
Age	1.039 (1.031, 1.047)	<0.001**	1.020 (1.010, 1.031)	<0.001**	1.010 (1.002, 1.018)	0.010**	1.001 (0.994, 1.008)	0.841
Gender		0.171		0.101		0.183		<0.001**
Female	1		1		1		1	
Male	1.126 (0.950, 1.334)		1.220 (0.962, 1.546)		1.132 (0.943, 1.359)		1.685 (1.421, 1.997)	
Smoking		0.168		0.635		0.360		0.969
No	1		1		1		1	
Yes	1.148 (0.944, 1.396)		1.067 (0.816, 1.397)		1.101 (0.896, 1.353)		0.996 (0.826, 1.202)	
Dialysis		<0.001**		0.134		0.546		0.628
No	1		1		1		1	
Yes	1.589 (1.275, 1.981)		0.733 (0.489, 1.100)		0.919 (0.698, 1.210)		1.056 (0.847, 1.316)	
Bladder cancer history		0.032*		0.093		0.018*		<0.001**
No	1		1		1		1	
Yes	1.209 (1.016, 1.440)		1.237 (0.965, 1.586)		1.251 (1.038, 1.506)		1.618 (1.368, 1.914)	
Tumor location		0.102		0.629		0.692		0.001**
Renal pelvis	1		1		1		1	
Ureter	1.178 (0.987, 1.406)	0.070	1.121 (0.868, 1.448)	0.380	1.057 (0.872, 1.281)	0.575	1.338 (1.124, 1.591)	0.001**
Synchronous	1.221 (0.959, 1.556)	0.106	1.108 (0.802, 1.529)	0.534	1.105 (0.861, 1.419)	0.431	1.417 (1.097, 1.831)	0.008**
Surgical approach		0.003**		0.023*		0.540		0.616
Open	1		1		1		1	
Laparoscopy	0.795 (0.684, 0.925)		0.784 (0.635, 0.967)		0.950 (0.808, 1.118)		1.042 (0.887, 1.225)	
Pathological T stage		<0.001**		<0.001**		<0.001**		0.010**
pTis/pTa	1		1		1		1	
pT1	1.197 (0.892, 1.605)	0.230	1.280 (0.627, 2.614)	0.498	1.154 (0.756, 1.760)	0.507	1.285 (1.017, 1.624)	0.036*
pT2	1.439 (1.059, 1.955)	0.020*	3.325 (1.712, 6.457)	<0.001**	2.561 (1.716, 3.821)	<0.001**	1.223 (0.943, 1.588)	0.130
pT3	2.806 (2.106, 3.739)	<0.001**	8.644 (4.576, 16.326)	<0.001**	5.072 (3.464, 7.427)	<0.001**	1.214 (0.934, 1.578)	0.147
pT4	6.143 (4.295, 8.786)	<0.001**	17.867 (8.978, 35.560)	<0.001**	9.748 (6.290, 15.106)	<0.001**	0.467 (0.238, 0.914)	0.026*
Pathological N stage		<0.001**		<0.001**		<0.001**		0.632
pN0	1		1		1		1	
pNx	1.144 (0.956, 1.369)	0.143	1.122 (0.867, 1.452)	0.383	1.135 (0.935, 1.378)	0.199	1.081 (0.909, 1.287)	0.377
pN+	2.658 (2.018, 3.501)	<0.001**	2.825 (2.017, 3.955)	<0.001**	2.604 (1.992, 3.403)	<0.001**	0.977 (0.638, 1.498)	0.917

SUPPLEMENTARY TABLE Continued

Multivariate analysis	OS		CSS		DFS		BRFS	
	HR (95% CI)	*p* value	HR (95% CI)	*p* value	HR (95% CI)	*p* value	HR (95% CI)	*p* value
Tumor grade		0.008**		0.002**		0.003**		0.453
Low grade	1		1		1		1	
High grade	1.421 (1.097, 1.841)		2.516 (1.408, 4.493)		1.659 (1.183, 2.328)		0.924 (0.753, 1.135)	
Multifocality		0.223		0.086		0.008**		0.601
No	1		1		1		1	
Yes	1.139 (0.924, 1.405)		1.288 (0.964, 1.721)		1.345 (1.079, 1.677)		1.058 (0.856, 1.307)	
Postoperative chemotherapy		0.888		0.478		0.292		0.587
No	1		1		1		1	
Yes	1.014 (0.836, 1.229)		0.916 (0.720, 1.167)		1.106 (0.917, 1.334)		1.063 (0.853, 1.326)	
Gross hematuria		0.016*		0.048*		0.260		0.614
No	1		1		1		1	
Yes	0.811 (0.683, 0.962)		0.791 (0.627, 0.998)		0.900 (0.749, 1.081)		0.954 (0.796, 1.144)	
Symptomatic hydronephrosis		0.001**		<0.001**		0.002**		0.151
No	1		1		1		1	
Yes	1.386 (1.150, 1.694)		1.599 (1.239, 2.064)		1.389 (1.131, 1.705)		0.843 (0.668, 1.064)	

CI, confidence interval; HR, hazard ratio; OS, overall survival; CSS, cancer-specific survival; DFS, disease-free survival; BRFS, bladder recurrence-free survival.
* < 0.05, ** < 0.01
